# Supplementary material for: First Report of Genetic Variability of Erysipelothrix sp. Strain 2 in Turkeys Associated to Vero Cells Morphometric Alteration
Source: Pathogens. 2021 Feb 1;10(2):141. doi: 10.3390/pathogens10020141 (PMC7912226; doi:10.3390/pathogens10020141)
Supplement: Supplementary file 1 [file pathogens-10-00141-s001.pdf]

pathogens

MDPI

Supplementary Material

Table S1. Summary of the number of collections, samples and age in negative and positive ES2 flocks selected for the present study.

	Age of material collection	Number of collections	Number of organs	Number of samples	Number of positive samples
A	102 e 123	2	7	14	5
B	88,100,126	3	7	21	7
C	116	1	7	7	2
D	210	1	7	7	5
E	73 e 139	2	7	14	3
F	145	1	7	7	2
G	141	1	7	7	4
H	84	1	7	7	5
I	62 e 144	2	7	14	0
J	82	1	7	7	0
K	85, 94, 103, 112	4	7	28	0
L	107	1	7	7	0
M	115	1	7	7	0
N	88	1	7	7	0
O	88	1	7	7	0
P	109	1	7	7	0
Q	88	1	7	7	0
R	100	1	7	7	0

We studied only flocks in animals in final phase systems and breeder turkeys because no outbreaks have occurred in animals in the breeding phase. One flock was considered positive if at least one sample had *Erysipelothrix* sp strain 2 isolation.

Table S2. Mean mortality and age of the flocks that were positive and negative to ES2 at the outbreak.

	Positive-*Erysipelothrix sp.* strain 2	Negative-*Erysipelothrix sp.* strain 2	*p*-value
**Mean of final mortality**	13.11%	6.48%	0.0448
**Mean age of outbreak (days)**	120.6	94,57	<0.0001

Table S3. Distribution of the presence and absence of ES2 by age and organs in the flocks selected for the present study.

Flock	Type of creation	Number of isolates	*Erysipelothrix* sp. strain 2	Swine (to 7km)	Final Mortality (%)	Age (Days)	Organ
A	broiler turkeys	Est01	Pres	0	19.82	123	Liver
A	broiler turkeys	Est02	Pres	0	19.82	123	Heart
A	broiler turkeys	Est03	Pres	0	19.82	123	Spleen
A	broiler turkeys	Est04	Pres	0	19.82	123	Lung
A	broiler turkeys	there are no*	Pres	0	19.82	102	Spleen
B	broiler turkeys	there are no*	Pres	0	11.09	88	Liver
B	broiler turkeys	there are no*	Pres	0	11.09	100	Heart
B	broiler turkeys	there are no*	Pres	0	11.09	100	Lung
B	broiler turkeys	there are no*	Pres	0	11.09	100	Liver
B	broiler turkeys	there are no*	Pres	0	11.09	100	Kidney
B	broiler turkeys	Est05	Pres	0	11.09	126	Liver
B	broiler turkeys	Est7	Pres	0	11.09	126	Kidney
C	broiler turkeys	Est6	Pres	0	12.54	116	Spleen
D	Breeding turkeys	Est8	Pres	1	13.33	210	Lung
D	Breeding turkeys	Est9	Pres	1	13.33	210	Lung
D	Breeding turkeys	Est10	Pres	1	13.33	210	Liver
D	Breeding turkeys	there are no*	Pres	1	13.33	210	Spleen
D	Breeding turkeys	Est11	Pres	1	13.33	210	Kidney

E	broiler turkeys	Est12	Pres	1	12.29	73	Lung
E	broiler turkeys	Est13, Est14	Pres	1	12.29	139	Liver
E	broiler turkeys	Est15, Est16	Pres	1	12.29	139	Heart
C	broiler turkeys	Est17	Pres	0	12.54	116	liver
F	broiler turkeys	Est18	Pres	0	17.25	145	Spleen
F	broiler turkeys	Est19	Pres	0	17.25	145	Liver
G	broiler turkeys	Est20	Pres	1	7.93	141	Spleen
G	broiler turkeys	Est21	Pres	1	7.93	141	Lung
G	broiler turkeys	Est22	Pres	1	7.93	141	Heart
G	broiler turkeys	Est23	Pres	1	7.93	141	Liver
H	broiler turkeys	Est24	Pres	1	10.62	84	Spleen
H	broiler turkeys	Est25, Est30	Pres	1	10.62	84	Heart
H	broiler turkeys	Est26	Pres	1	10.62	84	Lung
H	broiler turkeys	Est27, Est28	Pres	1	10.62	84	Liver
H	broiler turkeys	Est29	Pres	1	10.62	84	Kidney
I	broiler turkeys	no	Absent	0	7.83	62	Absent in all organs
J	broiler turkeys	no	Absent	0	6.87	82	Absent in all organs
K	broiler turkeys	no	Absent	0	6.03	100	Absent in all organs
L	broiler turkeys	no	Absent	1	5.39	107	Absent in all organs
M	broiler turkeys	no	Absent	0	7.38	115	Absent in all organs
N	broiler turkeys	no	Absent	0	4.96	88	Absent in all organs
O	broiler turkeys	no	Absent	0	6.28	88	Absent in all organs
P	broiler turkeys	no	Absent	0	7.27	109	Absent in all organs
Q	broiler turkeys	no	Absent	0	6.8	35	Absent in all organs
R	broiler turkeys	no	Absent	0	5.99	100	Absent in all organs

H and L: This is the same aviary but flock in different production cycle; *there are no: Strains were not saved; In positive flocks we show just positive results. Other sample and organs are negatives.

Table S4. Association between positive sample to ES2 and the presence of swine flocks (maximum 7Km distance).

	**Positive ES2**	**Negative ES2**
Swine flocks (7Km)	17	7
No swine flocks	16	142

$p < 0.0001$; OR = 21.55. As in the same poultry farm near the rearing of pigs, there was positivity for ES2 in one cycle and negativity in the next cycle; we calculated the OR based on the number of samples.

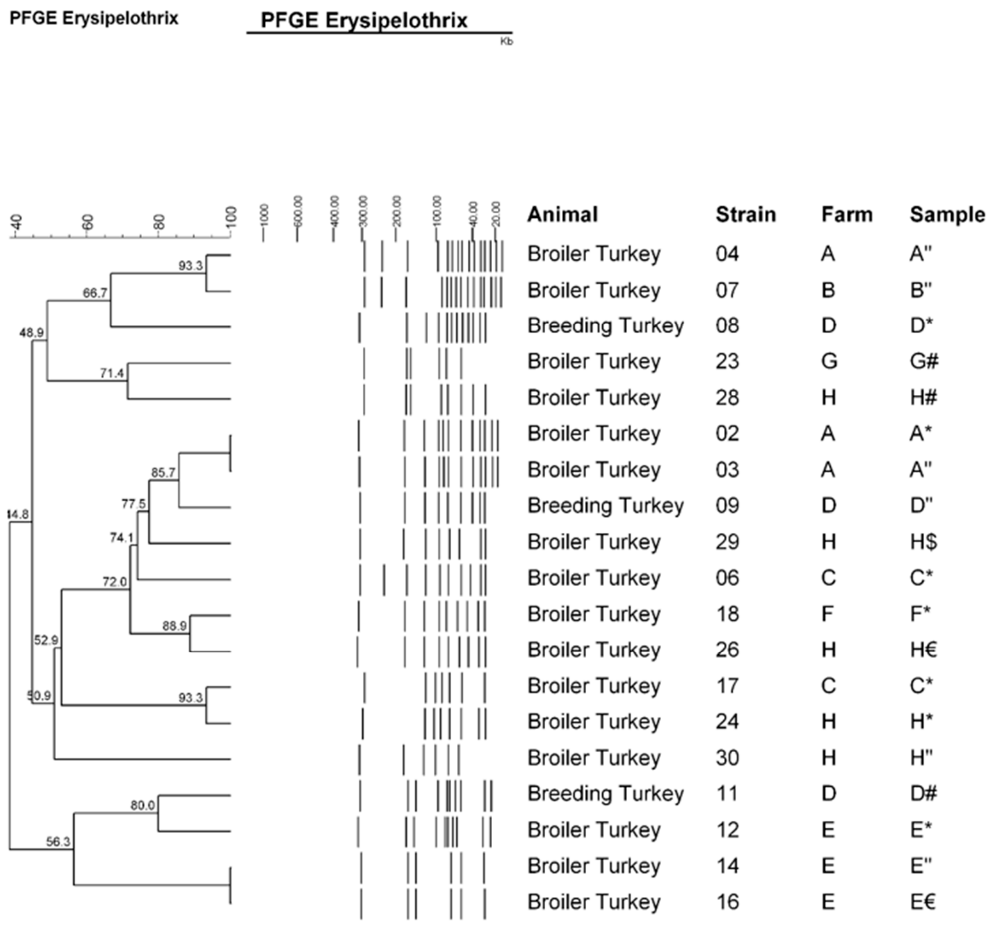

Figure S1. Bands profile found in the PGFE analysis of 19 strains *Erysipelothrix* sp strain 2 isolated from turkeys.

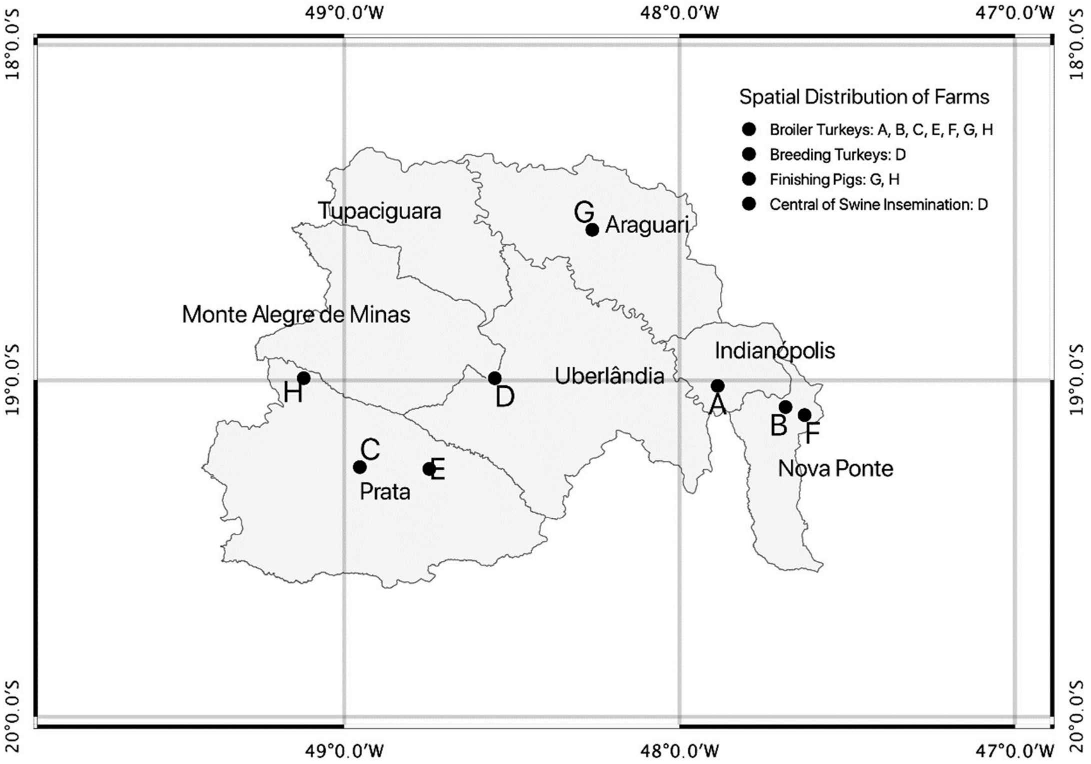

Figure S2. Georeferenced location of positive turkey and swine farms.

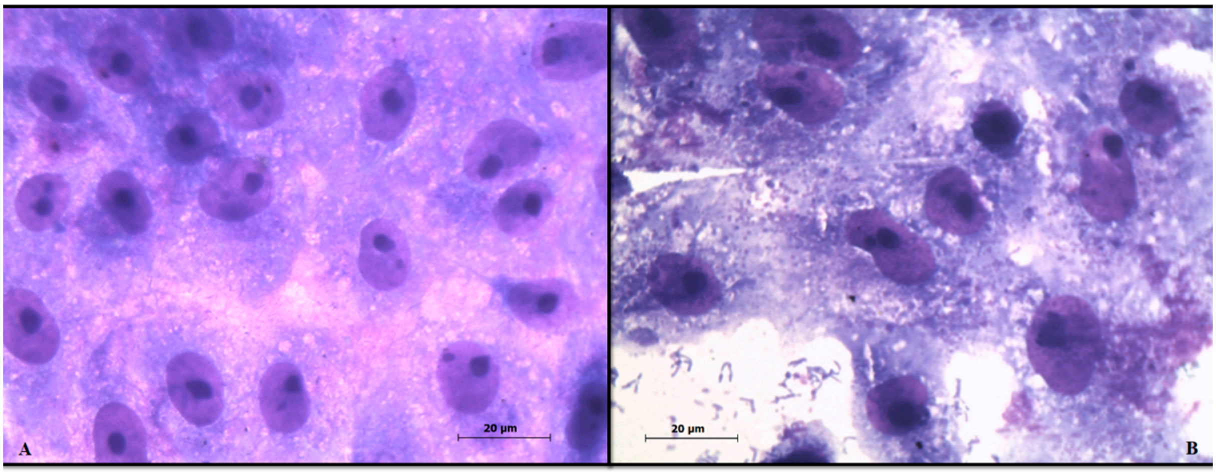

Figure S3. Vero cells stained with panoptic dye. A. Vero Cell control group at 4 hours of analysis (1000-fold increase). B. Vero Cell D0 Est06 group at 4 hours after inoculation (1000-fold increase).
